# Major histocompatibility complex class II deficiency in Morocco: a 26-year retrospective cohort study

**DOI:** 10.3389/fimmu.2026.1831840

**Published:** 2026-08-24

**Authors:** Aziza Bachir Kattra, Fatima Ailal, Ibtihal Benhsaien, Noureddine Rada, Brahim Admou, Raja Hazime, Noufissa Benajiba, Naima El Hafidi, Mohamed Hbibi, Moustapha Hida, Maria Rkain, Yousra El boussaadni, Abdellah Oulmaati, Ahmed Aziz Bousfiha, Jalila El Bakkouri

**Affiliations:** 1 Laboratory of Clinical Immunology, Infection, and Autoimmunity (LICIA), Faculty of Medicine and Pharmacy, Hassan II University, Casablanca, Morocco; 2 Department of Pediatrics Infectious and Immunological Diseases, Abderrahim El Harouchi Mother-Child Hospital, University Hospital Center Ibn Rochd, Casablanca, Morocco; 3 Department of Pediatric, Mohammed VI University Hospital, Marrakech, Morocco; 4 Laboratory of Immunology, Center of Clinical Research, Mohammed VI University Hospital, Faculty of Medicine and Pharmacy, Cadi Ayyad University, Marrakech, Morocco; 5 Department of Pediatrics, Abderrahim El Harouchi Mother-Child Hospital, University Hospital Center Ibn Rochd, Casablanca, Morocco; 6 Department of Pediatric Immuno-Allergology and Infectious Diseases, Children Hospital, Ibn Sina University Hospital, Rabat, Morocco; 7 Department of Pediatric Hematology-Oncology SHOP, Hassan II University Hospital, Fez, Morocco; 8 Department of Pediatrics, Hassan II Hospital, Fez, Morocco; 9 Department of Pediatrics, Centre Hospitalier Universitaire (CHU) Mohamed VI, Mohamed First University, Oujda, Morocco; 10 Department of Pediatrics, Mohammed VI University Hospital Center, Tanger, Morocco; 11 Immunology Laboratory, University Hospital Center Ibn Rochd, Casablanca, Morocco; 12 Immunopathology, Immunomonitoring, and Immunotherapy Laboratory, Mohammed VI University of Health Sciences (UM6SS), Casablanca, Morocco

**Keywords:** CIITA, combined immunodeficiency, hematopoietic stem cell transplantation, HLA-DR expression, MHC-II deficiency, Morocco, RFXANK mutation

## Abstract

Major histocompatibility complex class II (MHC-II) deficiency is a rare autosomal recessive combined immunodeficiency caused by defects in transcriptional regulators controlling HLA class II expression. The disorder is more prevalent in North Africa due to high rates of consanguinity and founder effects. Updated national data remain limited (in Morocco, up-to-date national data on this deficit remain limited). We aimed to describe the epidemiological, clinical, immunological, and genetic characteristics, as well as the outcomes of Moroccan patients with MHC-II deficiency. We conducted a retrospective cross-sectional study including 54 patients diagnosed with MHC-II deficiency at the national referral center for primary immunodeficiencies in Casablanca, between January 1998 and December 2024. The diagnosis was based on the absence or marked reduction (<5%) in HLA-DR surface expression on B lymphocytes and monocytes, as demonstrated by flow cytometry, with molecular confirmation when available. Demographic, clinical, immunological, genetic, therapeutic, and outcome data were analyzed. Included patients were issued from 47 unrelated families, and parental consanguinity was reported in 87% of cases. Median age at symptom onset was 6 months and median age at diagnosis was 19 months, reflecting a diagnostic delay of 13 months. Respiratory infections were the most frequent manifestation (96%), followed by gastrointestinal involvement (68.5%), mucocutaneous infections (75.9%), and failure to thrive (96%). Autoimmune cytopenia occurred in 20.4% of patients. Immunologic evaluation showed CD4^+^ T-cell lymphopenia in 86.7%, with hypogammaglobulinemia. Genetic testing (*n* = 35) identified the recurrent RFXANK founder deletion (c.338-25_338del26) in 97.1% of genotyped patients, while one patient carried a CIITA mutation. Hematopoietic stem cell transplantation (HSCT) was performed in eight patients (14.8%), with a survival rate of 62.5% among transplanted cases. Overall mortality reached 66.7%. MHC-II deficiency in Morocco is characterized by early onset, high consanguinity, and a strong RFXANK founder effect, but remains marked by delayed diagnosis and poor survival. Early clinical recognition, targeted molecular screening in high-risk families, improved access to HSCT, and optimized preventive strategies are critical to improve outcomes.

## Introduction

Major histocompatibility complex class II (MHC-II) deficiency, also called bare lymphocyte syndrome type II, is a rare autosomal recessive combined immunodeficiency defined by absent or markedly reduced expression of HLA class II molecules DR, DQ, and DP on antigen-presenting cells. The defect arises from variants in four transcriptional regulators of MHC-II rather than in structural HLA genes, namely, the Class II trans-activator CIITA and the RFX complex proteins RFXANK, RFX5, and RFXAP (1–3). Loss of MHC-II expression impairs positive thymic selection of CD4^+^ T cells and limits helper T-cell function in the periphery, producing profound CD4^+^ lymphopenia and defective T-cell-dependent immune responses (2, 3). Most patients experience recurrent and often opportunistic infections of viral, bacterial, fungal, or protozoal origin during their first year of life, with chronic diarrhea, failure to thrive, and features of immune dysregulation (3, 4). Without a definitive intervention, survival beyond early childhood is uncommon (5). Hematopoietic stem cell transplantation (HSCT) is the only curative option, and outcomes have improved with better conditioning, donor selection, and infection control, but pre-existing infection and organ damage at diagnosis remain major determinants of survival, which underlines the need for early recognition and referral (5–7).

Approximately 200 to 250 cases have been reported worldwide (3, 8–21). About two-thirds arise in consanguineous families from North Africa and the Middle East, where some founder mutations are well known (12–21). In North African cohorts, a recurrent 26-bp deletion in RFXANK, c.338-25_338del26, was detected in approximately 70% to 90% of genotyped patients (12–18). However, recent reports have identified variants in CIITA and in other RFX complex genes, indicating a broader genetic spectrum than previously thought (8, 18–22). Underdiagnosis remains substantial in the region as clinical recognition varies, access to immunophenotyping and sequencing is limited, and national primary immunodeficiency (PID) registries are likely to capture only a small fraction of the actual burden (23). In Morocco, an initial study identified the RFXANK founder mutation in a cohort of 10 patients and highlighted diagnostic delay in some children (16). More recent observations have described cases extending beyond early childhood (17), and rare cases of CIITA deficiency (22).

In this background, North African series remain essential to define the clinical spectrum and genetic landscape of MHC-II deficiency, yet updated multicenter or long-horizon single-center cohorts are still needed. The present study reports a 26-year experience from a national referral center in Casablanca and describes the epidemiologic, clinical, immunological, and molecular features, and HSCT outcomes of the largest cohort of Moroccan patients with MHC-II deficiency, placing these findings in the context of regional and international cohorts.

## Materials and methods

### Study design and setting

We conducted a retrospective cross-sectional, and single-center study of consecutive patients with MHC-II deficiency, enrolled between January 1998 and December 2024. Cases were diagnosed and followed up at the national referral center for PID/inborn errors of immunity (IEI) in Morocco, the Department of Clinical Immunology and Pediatric Infectious Diseases, Abderrahim Harouchi Mother–Child Hospital, University Hospital Centre CHU Ibn Rochd, Casablanca. Clinical and laboratory data were extracted from hospital records and laboratory databases using a standardized template. This study was based on patients with MHC class II deficiency identified from the Moroccan National Primary Immunodeficiency Registry. Some of these patients were previously included in the first national registry report published by our group (31); however, the present study represents a disease-specific analysis focusing exclusively on MHC class II deficiency, with updated clinical, immunological, genetic, and outcome data.

### Participants and case definition

Patients with an immunoclinical phenotype consistent with combined immunodeficiency and whose diagnosis of MHC-II deficiency was established by flow cytometry and/or genetic testing were recruited in this study. Case definition adhered to the European Society for Immunodeficiencies (ESID) clinical working definitions for IEI (24). The primary inclusion criterion was the absence or a marked reduction (<5%) of HLA-DR expression on circulating B lymphocytes, identified using either CD19 or CD20 according to reagent availability, and on CD14-positive monocytes, as assessed by flow cytometry. Human immunodeficiency virus (HIV) infection was excluded in all patients by routine serological testing.

### Data collection

The following parameters were recorded: age at symptom onset, age at diagnosis, gender, parental consanguinity, clinical manifestations by organ system, microbiological data, immunophenotyping analysis, immunoglobulin quantization, treatments, and outcomes. Data were implemented into a secure database with predefined quality checks, and discrepant entries were reconciled by two investigators.

### Immunologic assessment

Lymphocyte subsets were measured by flow cytometry on a BD FACSCanto II multi-laser cytometer (405, 488, and 633/640 nm) and analyzed with FACSDiva 8.0.1 software (both BD Biosciences). The antibody panel included CD45 V500, CD20 PE-Cy7, and HLA-DR PE. Isotype-matched control antibodies (BD Biosciences) were used to distinguish non-specific background from specific staining. Gating was performed using CD45 versus side scatter to identify lymphocytes and monocytes. B lymphocytes were identified using either CD19 or CD20, according to reagent availability, on the corresponding fluorescence versus side scatter plot. HLA-DR expression was then assessed on gated B lymphocytes and, when B-cell counts were markedly reduced, on CD14-positive monocytes. Serum immunoglobulins were measured by nephelometry according to the manufacturer’s instructions. Results were reported as absolute counts and as proportions according to age-related reference values.

### Microbiological investigations

Following clinical indications and local protocols, microbiological testing included culture and targeted molecular assays. For respiratory disease, viral polymerase chain reaction (PCR) was performed when indicated, including cytomegalovirus among others.

### Genetic analysis

Based on clinical orientation and immunophenotyping results, genetic confirmation targeted the four trans-acting regulators of MHC class II transcription: CIITA, RFXANK, RFX5, and RFXAP. Founder screening for the recurrent RFXANK c.338-25_338del26 deletion used conventional PCR followed by agarose gel electrophoresis for size discrimination. Primers flanked the deletion breakpoint (forward 5′-TTGGCAGCACTGGGGATAG-3′, reverse 5′-CCAGCAGACACAGCCAAAAC-3′). When founder screening was negative or the phenotype was atypical, targeted next-generation sequencing or gene-panel testing for IEI was performed according to availability. Parental samples were tested when available to confirm autosomal-recessive inheritance.

### Management and outcomes

Supportive management comprised antimicrobial treatment of acute infections, antimicrobial prophylaxis based on clinical risk, and intravenous immunoglobulin replacement when indicated. HSCT was offered when a suitable donor was identified and the patient was clinically stable. Transplant variables included donor type, conditioning approach, and early infectious complications. The primary criteria of outcomes recorded were survival status at the last follow-up and the time between diagnosis and death or last contact. Secondary outcomes included the frequency of major infection categories, immune cytopenia and other immune dysregulation, and radiologic complications such as bronchiectasis. For transplanted patients, transplant survival at last follow-up was recorded.

### Statistical analysis

Data were compiled in a structured database and analyzed with standard statistical software. Continuous variables are presented as means with standard deviations or as medians with ranges. Categorical variables are presented as counts with percentages. Where data permitted, survival was described with time-to-event methods. Given the retrospective design and cohort size, analyses are descriptive.

### Ethics

The study complied with institutional and national standards and the Declaration of Helsinki. Ethics approval was granted by the Ibn Rochd University Hospital ethics committee under approval number 2020/DoEHRSI/039. Informed consent for diagnostic procedures and for the use of anonymized data was obtained from parents or legal guardians for minors and from adult patients when applicable.

## Results

### Cohort and demographics

A total of 54 patients with MHC-II deficiency from 47 unrelated families were included in the study. Among these, six were multiplex families comprising 13 affected siblings. Patients were referred from various regions across Morocco. Parental consanguinity was documented in 47 out of 54 cases (87%). The median age at symptom onset was 6 months, while the median age at diagnosis was 19 months (range: 5 days to 26 years), resulting in a median diagnostic delay of 13 months. This diagnostic delay likely reflects both limited clinical recognition of MHC class II deficiency and the absence of a neonatal screening program for this disorder in Morocco. The sex distribution was balanced, with a male-to-female ratio of 1.07. The summary of epidemiological and survival characteristics is detailed in **Table 1**.

**Table 1:** Epidemiological and survival characteristics of patients with MHC-II deficiency included in this study.

Characteristic	Value
Total patients	54
Families represented	47
Multiplex families	6
Affected siblings in multiplex families	13
Consanguinity	47/54 (87%)
Age at symptom onset, median	6 months
Age at diagnosis, median (range)	19 months (5 days–26 years)
Male-to-female count (ratio)	28:26 (1.07)
Underwent HSCT	8/54 (14.8%)
Alive at last follow-up	15/54 (27.8%)
Deceased	36/54 (66.7%)
Lost to follow-up	3/54 (5.6%)

### Clinical manifestations

Respiratory infections were the most common and were documented in 52 cases (96.3%), of which 40 cases (74.1%) had pneumonia, and 12/54 (22.2%) had bronchiectasis on follow-up imaging. Gastrointestinal symptoms were recorded in 37/54 (68.5%), dominated by chronic diarrhea, observed in 34 cases (63.0%). Mucocutaneous manifestations were present in 41/54 (75.9%). Ear, nose, and throat disease was noticed in 16 out of 54 cases (29.6%), namely, otitis media (13 cases, 24.1%) and sinusitis (3 cases, 5.6%). Urinary tract infections occurred in 10/54 (18.5%). Autoimmune cytopenias were observed in 11 cases (20.4%), namely, neutropenia (6 cases, 11.1%), autoimmune hemolytic anemia (3 cases, 5.6%), and thrombocytopenia (2 cases, 3.7%). Less common features included BCG-related disease and severe varicella, with 2 cases each (3.7%), and onychomycosis, with 1 case (1.9%), while failure to thrive was noted in 52 cases (96.0%). Full details are provided in **Table 2**.

**Table 2:** Clinical manifestations in Moroccan patients with MHC-II deficiency (*N* = 54).

Manifestation	n (%)
Pulmonary manifestations	52 (96.29)
Pneumonia	40 (74.07)
Bronchiectasis	12 (22.22)
Mucocutaneous manifestations	41 (75.92)
Oral candidiasis	27 (50.00)
Fungal dermatitis	6 (11.11)
Diaper erythema	3 (5.55)
Severe varicella	2 (3.70)
BCGitis	2 (3.70)
Onychomycosis	1 (1.85)
Digestive manifestations	37 (68.51)
Recurrent or chronic diarrhea	34 (62.96)
Esophageal candidiasis	3 (5.55)
Ear, nose, and throat (ENT) manifestations	16 (29.62)
Otitis media	13 (24.07)
Sinusitis	3 (5.55)
Autoimmune cytopenia	11 (20.37)
Neutropenia	6 (11.11)
Autoimmune hemolytic anemia	3 (5.55)
Thrombocytopenia	2 (3.70)
Urinary tract infections	10 (18.51)
Urinary tract infection	10 (18.51)
Other clinical manifestations	
Failure to thrive	52 (96.00)
Meningoencephalitis	4 (7.40)
Cow’s milk allergy	2 (3.70)
Hemophagocytic lymphohistiocytosis (HLH)	2 (3.70)

### Microbiology

Across 73 microbiological isolates, digestive sites contributed 32/73 (43.8%); pulmonary, 16/73 (21.9%); ear–nose–throat, 11/73 (15.1%); urinary, 8/73 (11.0%); and mucocutaneous, 6/73 (8.2%). The most frequent organisms overall were *Candida* species (17/73, 23.3%), predominantly from digestive and mucocutaneous samples, and cytomegalovirus in the respiratory tract (8/73, 11.0%). Bacterial isolates were diverse. *Salmonella* species were the leading enteric bacteria (8/73, 14.8%). *Pseudomonas aeruginosa* was recovered across multiple sites (6/73 combined). Full distributions by site are shown in **Table 3**.

**Table 3:** Microbiological isolates by site in Moroccan patients with MHC-II deficiency (*n* = 73).

Associated pathogens (n = 73)	N (%)
Pulmonary infections	16 (21.92)
* Cytomegalovirus*	8 (7.40)
* Coagulase-negative staphylococci*	3 (5.55)
* Streptococcus pneumoniae*	1 (1.60)
* Hemophilus influenzae*	1 (1.60)
* Pseudomonas aeruginosa*	1 (1.60)
* Rhinovirus/Enterovirus*	1 (1.60)
* Adenovirus*	1 (1.60)
Mucocutaneous infections	6 (8.22)
* Candida albicans*	2 (3.70)
* Pseudomonas aeruginosa*	1 (1.80)
* Morganella morganii*	1 (1.80)
* Proteus mirabilis*	1 (1.80)
* Viridans streptococci*	1 (1.80)
Digestive infections	32 (43.84)
* Candida* spp.	15 (27.77)
* Salmonella* spp. spp.	8 (14.81)
* Cryptosporidium (oocysts)*	2 (3.70)
* Entamoeba histolytica*	1 (1.80)
* Enterococcus* spp. spp.	1 (1.80)
* Enterococcus faecium*	1 (1.80)
* Escherichia coli + Klebsiella* spp. spp.	1 (1.80)
* Rotavirus*	1 (1.80)
* Blastocystis hominis*	1 (1.80)
* Giardia*	1 (1.80)
ENT infections	11 (15.07)
* Pseudomonas aeruginosa*	3 (3.70)
* Staphylococcus* spp. spp.	2 (3.70)
* Klebsiella pneumoniae*	2 (3.70)
* Acinetobacter baumannii*	1 (1.80)
* Enterobacter cloacae*	1 (1.80)
* Proteus mirabilis*	1 (1.80)
* Escherichia coli*	1 (1.80)
Urinary tract infections	8 (10.96)
* Escherichia coli*	4 (7.40)
* Klebsiella pneumoniae*	2 (3.70)
* Candida* spp. spp.	1 (1.80)
* Pseudomonas aeruginosa*	1 (1.80)

### Immunologic findings

**Figure 1:**
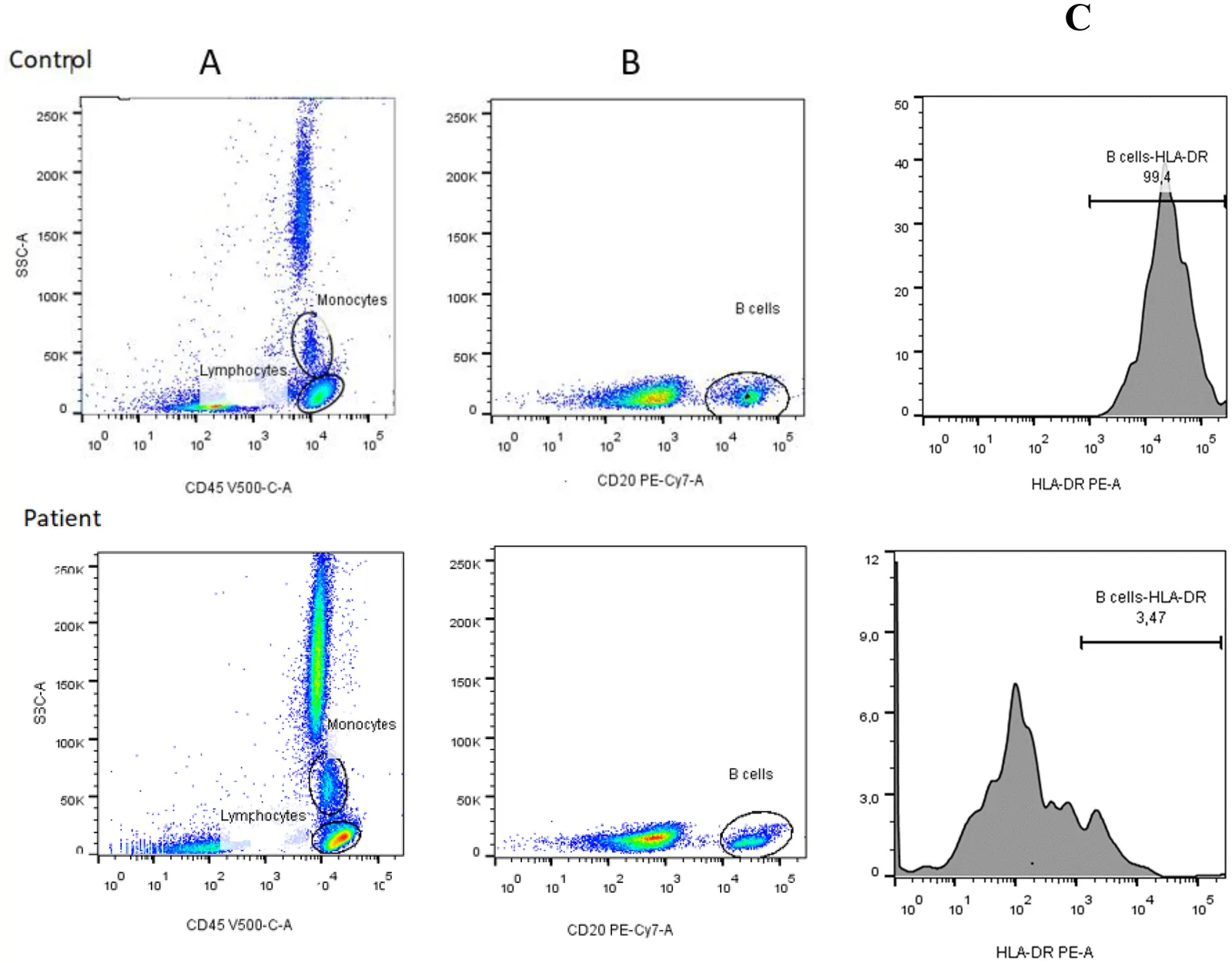
HLA-DR expression by flow cytometry in MHC-II deficiency. Representative plots from a healthy control (top) and an affected patient (bottom). **(A)** Gating of CD45^+^ lymphocytes and monocytes. **(B)** CD20^+^ B-cell gate. **(C)** HLA-DR expression on B cells. HLA-DR-positive B cells were 99.4% in the control and 3.47% in the patient; HLA-DR <5% was the diagnostic threshold. Acquisition on FACSCanto-II and analysis with FACSDiva 8.0.1 (both BD Biosciences). Isotype control antibodies (BD Biosciences) were used to define non-specific background.

**Figure 2:**
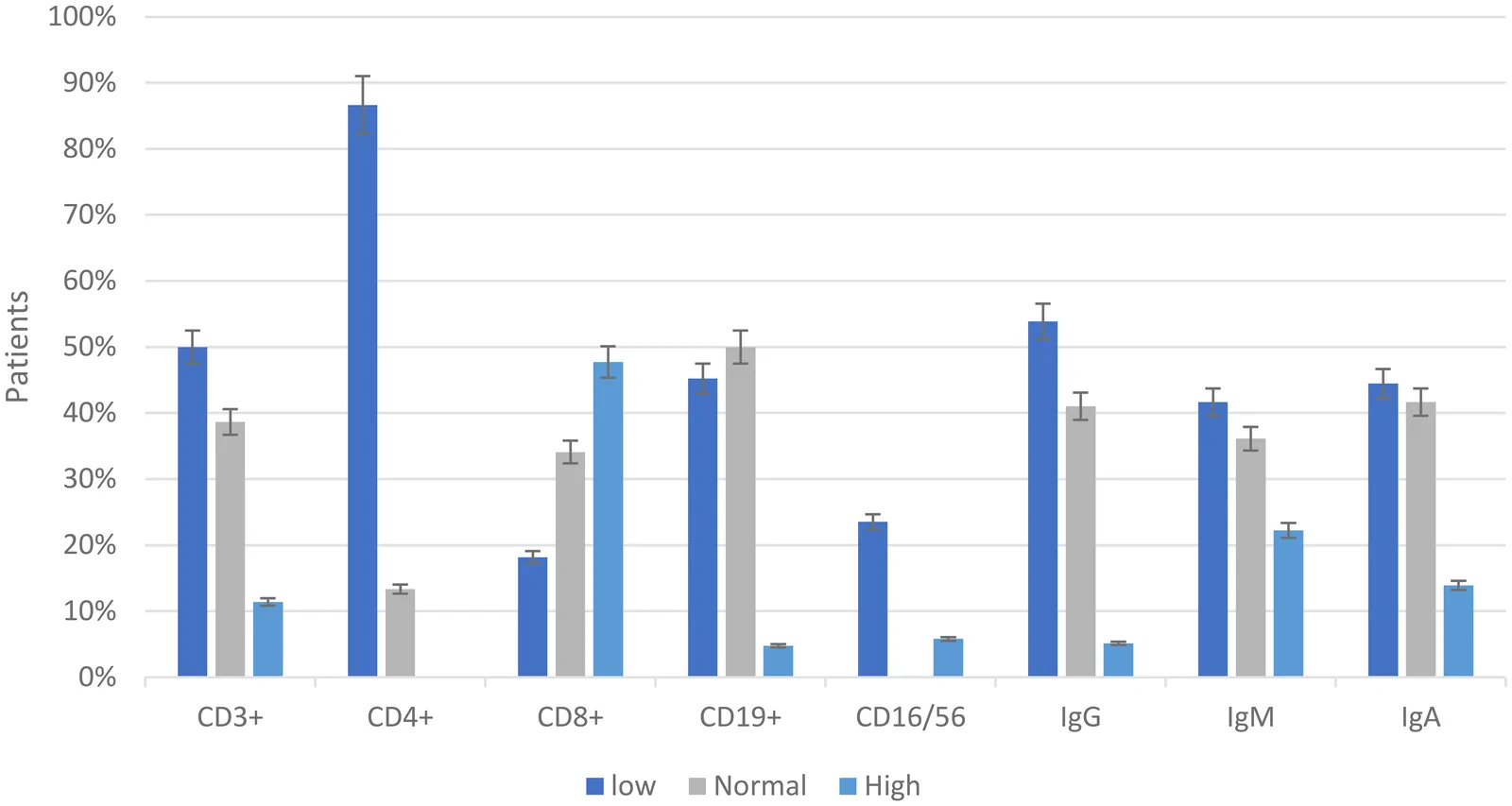
Distribution of lymphocyte subsets and serum immunoglobulins in the Moroccan cohort. According to levels of immunological values, patients are classified as low, within reference, or high for each marker using age-matched reference intervals. Bars show the percentage of patients. The most frequent abnormalities were low CD4^+^ T cells (*n* = 39, 86.7%), low IgG (*n* = 21, 53.8%), and low IgA (n = 16, 44.4%); IgM was within range in 13 cases (36.1%).

Complete immunologic data were available for 45 patients. HLA-DR expression on B cells or on monocytes was below 5% in all tested patients, consistent with MHC class II deficiency (**Figure 1**). CD4^+^ T-cell lymphopenia was present in 86.7% of cases (*n* = 39), and B-cell lymphopenia was noticed in 45.2% of cases (*n* = 19). Serum immunoglobulins were reduced for IgG in 21 cases (53.8%) and IgA in 16 cases (44.4%), while IgM was within the reference range in 13 cases (36.1%). Distributions of lymphocyte subsets and serum immunoglobulin levels for the cohort are shown in **Figure 2**.

### Genetic findings

**Figure 3:**
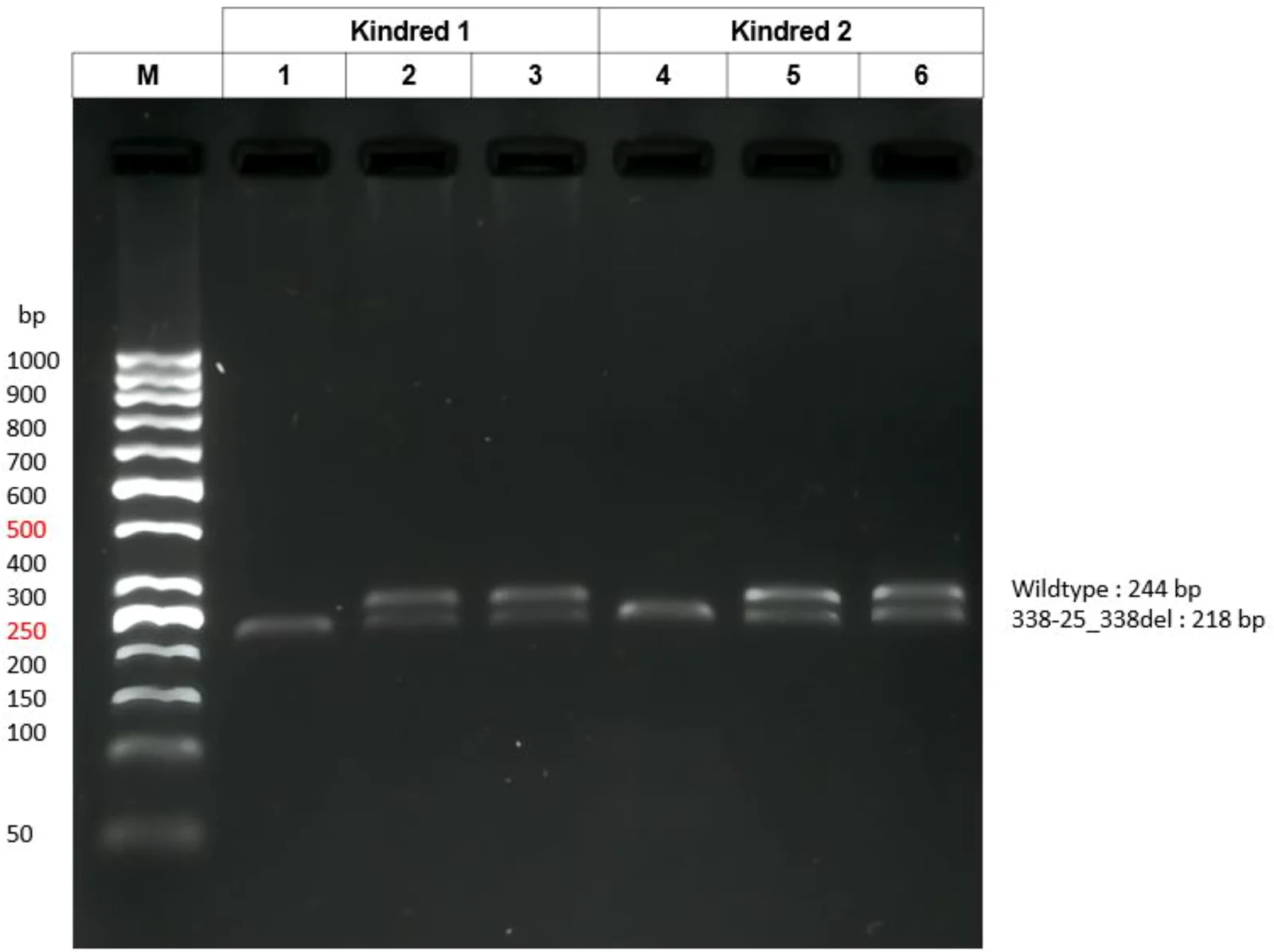
PCR detection of the RFXANK founder deletion (c.338-25_338del26). Representative gel showing the wild-type band at 244 bp and the deletion band at 218 bp in two kindreds. Lane M, molecular size ladder. Kindred 1: lanes 1–3: proband homozygous deletion (218 bp), mother heterozygous (244/218 bp), and father heterozygous (244/218 bp). Kindred 2: lanes 4–6: proband homozygous deletion (218 bp), mother heterozygous (244/218 bp), and father heterozygous (244/218 bp). Across the cohort, 34/35 genotyped patients (97.1%) carried the homozygous RFXANK c.338-25_338del26 deletion.

Genetic testing was performed in 35/54 (64.8%) patients. Among genotyped patients, the recurrent RFXANK c.338-25_338del26 deletion was identified in 34/35 (97.1%), all homozygous. One patient had a homozygous CIITA c.1615C>T (p.Arg539*) variant. Parental testing, when available, showed heterozygosity consistent with autosomal-recessive inheritance. Overall, at least 34/54 (63.0%) of the total cohort had a confirmed RFXANK founder deletion. Gel electrophoresis results and family studies for two representative kindreds are shown in **Figure 3**.

### Treatment and outcomes

All patients received regular intravenous immunoglobulin and sulfamethoxazole–trimethoprim (co-trimoxazole) prophylaxis according to clinical need. Eight patients (14.8%) underwent HSCT. The donors were siblings, HLA-identical in five cases (62.5%) and haploidentical in three cases (37.5%); one patient underwent two haploidentical transplant procedures at the ages of 7 and 9. Patients received a conditioning regimen consisting of busulfan (3.2 mg/kg/day for 2 days) and fludarabine (30 mg/m²/day for 5 days, from day −6 to day −2). Graft-versus-host disease prophylaxis consisted of cyclosporine administered for 3 to 6 months following HSCT. The time from diagnosis to first HSCT ranged from 12 to 69 months with a median of 37.5 months. Five transplanted patients (62.5%) were alive at the last follow-up, with a median age of 17 years (range 10–24), while the three other patients died following severe infections, including cytomegalovirus, *Candida albicans* and *Salmonella enteritidis* septicemia, with a median age at death of 48 months (range, 9 months–4.5 years). Among non-transplanted patients, 36 died during follow-up. Ten patients aged between 1 month and 17 years, with an average age of 8.12 years and a median age of 5 years, are still alive and are being monitored regularly in our department, while three patients were lost to follow-up.

Transplant characteristics and outcomes are summarized in **Table 4**.

**Table 4:** Hematopoietic stem cell transplantation (HSCT) in Moroccan patients with MHC-II deficiency.

No.	Sex	Age at diagnosis (months)	Age at HSCT (years)	Donor type	Outcome	Age at last contact/death	Cause of death (if applicable)	Time from diagnosis to first HSCT** (months)
1	F	18	ND	Haploidentical	Deceased	4.5 years	Invasive *Candida albicans*	ND
2	M	8	ND	Haploidentical	Deceased	9 months	Cytomegalovirus infection	ND
3	F	72	ND	HLA-identical sibling	Alive	24 years	—	ND
4	F	15	7	HLA-identical sibling	Alive	17 years	—	69
5	M	15	4	HLA-identical sibling	Deceased	4 years	Septicemia	33
6	F	36	4	HLA-identical sibling	Alive	21 years	—	12
7	F	ND	2.5	HLA-identical sibling	Alive	10 years	—	ND
8	M	42	7; 9*	Haploidentical (two HSCTs)	Alive	12 years	—	42

ND, not documented. *Where two HSCTs occurred, both ages are shown. **Time from diagnosis to first HSCT is derived when both ages at diagnosis and HSCT are available.

## Discussion

Our cohort of 54 Moroccan patients with MHC-II deficiency represents one of the largest single-country series to date. It highlights the marked geographical clustering of MHC-II deficiency in the Middle East and North Africa (MENA) region, which collectively account for more than two-thirds of globally reported cases, while paradoxically underscoring the scarcity of comprehensive published national series (3, 8–21). Regional registries confirm that MHC-II deficiency accounts for 4.1% of all IEI in the MENA region (702/17,120 cases) (25), compared with less than 1% in Europe (26) and Asia (27, 28). Strikingly, national registries show much higher proportions in Algeria (11.2%) (29), Tunisia (9.8%) (30), and Morocco (6.2%) (31), than in Western countries. This geographical contrast reflects the dual impact of high consanguinity and strong founder effects. Indeed, consanguinity was present in 87% of our cohort, a figure (picture or state) that mirrors the rates reported in Tunisia (~80%) (13), Turkey (90.5%) (9), and Iran (100%) (19). This may explain the nearly equal sex ratio in our cohort. Indeed, reported sex ratios vary slightly across studies, likely due to small sample sizes, with a Turkish single-center cohort reporting a female-to-male ratio of 1.63, compared with 1.18 in a large North African survey (9, 12). Across all series, the median age at onset of symptoms is consistently in early infancy, with 6 months in our cohort and in Tunisia, 5 months in Iran, and 10 months in Algeria, yet the age at molecular diagnosis is often delayed (13, 15, 19). In our series, the median diagnostic age was 19 months, reflecting a 13-month diagnostic lag, compared to 2.7 years in Algeria (15) and 8 months in Turkey (9), underscoring persistent delays across the region.

Autosomal recessive defects in RFXANK, RFX5, CIITA, or RFXAP are the currently known causes of MHC-II deficiency, with RFXANK mutations responsible for nearly two-thirds of reported cases worldwide and almost all patients in the North African series (12–18). The predominance of the RFXANK 752delG26 allele, detected in 97.1% of our series, 81.8% in Algeria, and 73.5% in Tunisia, further demonstrates the founder effect in North Africa (13, 15). By comparison, Iran exhibits a different founder mutation (RFXANK c.162delG) (19), and in non-Maghrebian populations, the genetic spectrum is more heterogeneous. In Turkey, RFXANK mutations accounted for 25.7% of cases and RFX5 accounted for 22.8%, with additional contributions from CIITA (11.4%) and RFXAP (5.7%) (8). Egyptian patients similarly displayed a mixed profile, with RFXANK mutations in 40% and RFX5 in 30%, alongside occasional CIITA defects (18). Smaller cohorts from China, India, Sudan, and North America further expand this heterogeneity, describing rare or compound heterozygous variants in all four regulatory genes. Collectively, these observations highlight both the major contribution of North African and Middle Eastern patients to defining the global epidemiology and molecular architecture of MHC-II deficiency, and the persistent limitations imposed by restricted access to broad genetic testing and reliance on founder-mutation screening, which continue to hamper comprehensive characterization across the region.

Clinically, our cohort exhibits a phenotype remarkably consistent with previously reported series from the MENA region, while providing important insights into disease burden and management challenges. Respiratory involvement was almost universal (96.3%, 52/54), with pneumonia in 74.1% (40/54) and bronchiectasis already established in 22.2% at diagnosis. Similar rates are reported in Tunisia (82.4%, 28/34) (13) and Turkey (81%), where 14% developed bronchiectasis during follow-up (9), while Algerian data note recurrent respiratory infections in 54.5% (6/11) (15). The high prevalence of bronchiectasis at presentation across these cohorts strongly suggests diagnostic delays and missed opportunities for early intervention. Gastrointestinal disease was also common (68.5%, 37/54), mainly chronic diarrhea (63.0%) and esophageal candidiasis (5.6%), with rates similar to Tunisian (76.5%), Turkish (77.1%, 27/35), and Algerian (90.9%) series (8, 13, 15). Failure to thrive was almost universal (96.0%), underscoring the cumulative burden of infections, diarrhea, and nutritional compromise. Nevertheless, the nutritional status of patients is not systematically assessed. Mucocutaneous fungal infections were frequent in our cohort (75.9%, 41/54), with oral candidiasis in 50%, exceeding a Turkish report (28.6%, 6/21) (9), but close to Algerian data (27.3%) (15). Opportunistic and vaccine-related complications, though less common, were clinically important: BCGitis and severe varicella each occurred in 3.7% (2/54), while regional reports describe oral poliovirus vaccine-associated acute flaccid paralysis in 33.3% (3/9) of Egyptian patients (18), and a fatal case of vaccine-associated poliomyelitis in Iran (19), emphasizing the importance of avoiding live vaccines when MHC-II deficiency is suspected. Immune dysregulation affected 20.4% (11/54), mainly neutropenia (11.1%) and autoimmune hemolytic anemia (5.6%), comparable to Turkish cohorts (19%) (9), confirming that autoimmunity, though less frequent, may be clinically relevant. These findings confirm the typical severe phenotype of MHC-II deficiency while highlighting persistent gaps in early recognition, nutritional assessment, and timely supportive care needed to prevent irreversible complications and improve long-term outcomes.

Immunologically, our cohort showed a specific profile of MHC-II deficiency, with CD4^+^ T-cell lymphopenia observed in 86.7% of patients, reflecting the critical requirement for class II expression on thymic epithelial cells for CD4 lineage selection. Comparable reductions have been reported in Tunisia (96%), Turkey (93.3%), Egypt (100%), and Algeria (81%), confirming the robustness of this hallmark (9, 13, 15, 18). Nevertheless, exceptions exist: in our series, six patients had normal CD4 counts despite complete absence of HLA-DR on B cells and monocytes, a finding also described in some patients, where residual or cell-type–restricted HLA-II expression may permit partial thymic selection yet fails to sustain effective peripheral T-cell help. Such cases emphasize that normal CD4 numbers do not exclude the diagnosis and may still carry a fatal infectious course. B-cell counts were normal in half of our patients and reduced in 45.2%, while serum immunoglobulins revealed selective class-switch defects, with IgG and IgA below age norms in approximately half, consistent with defective T–B collaboration rather than intrinsic B-cell failure. These findings connect with Turkish data, where IgG and IgA were reduced in >80% of cases despite preserved B-cell numbers in 88.5% (31/35) of cases (8). Crucially, HLA-DR expression was nearly absent in all tested Moroccan patients, confirming that flow cytometry of B cells and monocytes provides a sensitive frontline diagnostic marker. Taken together, the constellation of CD4^+^ lymphopenia, variable B-cell reduction, hypogammaglobulinemia, and absent HLA-DR expression defines a reproducible immunological triad that should trigger rapid genetic confirmation and early therapeutic planning.

Definitive cure of MHC-II deficiency requires HSCT, and outcomes in our cohort align with the broader experience. HSCT reconstitutes HLA-DR expression and CD4 T-cell function, but success rates have been variable. In the report of a North African cohort of RFXANK patients, 10 of 23 transplanted children (43%) were effectively cured with near-normal immune recovery (12). The Turkish analysis similarly showed dramatically better survival in transplanted patients: overall survival was only 28.6%, but among those who underwent HSCT, it rose to 80% (8). These data echo the pattern in Iran, where three of four transplanted patients survived, whereas the majority of non-transplanted patients died (19). In our Moroccan cohort, available data suggest that few patients have access to HSCT, which ties in with some Maghrebian reports, highlighting limited transplant resources. Nevertheless, those who do engraft often achieve long-term remission. Importantly, even without transplant, a subset of patients may survive into adulthood under intensive supportive care (12, 17). For example, Ouederni et al. noted seven transplanted‐unavailable patients (aged 6–32 years) who remain alive on immunoglobulin replacement and antibiotic prophylaxis (12). Thus, while HSCT should be pursued when feasible, aggressive infection prophylaxis and immunotherapy can extend survival in the interim. The main strength of this study lies in its scope, as it represents the largest national series of MHC-II deficiency in Morocco with follow-up extending over more than two decades. We combined detailed clinical, immunological, and genetic data, which allowed meaningful comparison with other regional cohorts. The use of uniform diagnostic criteria and a single-country setting also reduces heterogeneity.

However, several limitations should be acknowledged. As a retrospective study based on registry and hospital data, information was not always complete, and in some cases, full molecular confirmation was lacking. The absence of molecular testing in a subset of patients was mainly due to technical and financial constraints. Referral bias is also possible, as patients with more severe forms of the disease are more likely to be referred to our center. In addition, therapeutic approaches, including access to HSCT, have evolved over the study period, with access remaining limited, thereby restricting the interpretation of survival outcomes. Finally, comparison with other published series remains influenced by differences in methodology, case ascertainment, and follow-up. Despite these limitations, this study provides a comprehensive overview of MHC class II deficiency in Morocco and places our national experience within the broader international context.

## Conclusion

The current study highlights the key clinical and public health challenges of MHC-II deficiency. The most critical issue remains diagnostic delay, and although the disease onset occurs in early infancy, many patients are not diagnosed until irreversible complications such as bronchiectasis, malnutrition, or growth failure have already developed. This delay reflects both limited clinical awareness and the absence of newborn screening tailored to this disorder. Current T-cell receptor excision circles (TREC)-based assays used for severe combined immunodeficiency (SCID) screening do not detect MHC-II deficiency, further complicating early recognition. In countries with a high prevalence of founder mutations, targeted molecular approaches may offer a practical alternative. In Morocco, screening for the recurrent RFXANK 752delG26 allele in high-risk consanguineous families could enable earlier diagnosis, timely genetic counseling, and pre-symptomatic HSCT planning. Moreover, the risk of severe complications from live vaccines underscores the need for adapted immunization strategies, as illustrated by the successful Saudi experience with delayed neonatal BCG vaccination combined with SCID screening (32). Expanding access to molecular diagnostics, strengthening family counseling, and improving referral pathways are therefore essential to shorten diagnostic delays, prevent complications, and improve long-term outcomes in patients with MHC-II deficiency.

## Data Availability

Data sets are available on request: The raw data supporting the conclusions of this article will be made available by the authors, without undue reservation.
